# Exposure to cues associated with palatable food reward results in a dopamine D_2_ receptor-dependent suppression of evoked synaptic responses in the entorhinal cortex

**DOI:** 10.1186/1744-9081-9-37

**Published:** 2013-10-04

**Authors:** Juliana A Hutter, C Andrew Chapman

**Affiliations:** 1 Department of Psychology, Center for Studies in Behavioral Neurobiology, Concordia University, 7141 Sherbrooke Street W., Rm. SP-244, Montréal H4B 1R6, Québec, Canada

**Keywords:** Entorhinal cortex, Piriform cortex, Reward, Dopamine, Acetylcholine

## Abstract

**Background:**

The lateral entorhinal cortex receives inputs from ventral tegmental area dopamine neurons that are activated by exposure to food-related cues, and exogenously applied dopamine is known to modulate excitatory synaptic responses within the entorhinal cortex.

**Methods:**

The present study used *in vivo* synaptic field potential recording techniques to determine how exposure to cues associated with food reward modulates synaptic responses in the entorhinal cortex of the awake rat. Chronically implanted electrodes were used to monitor synaptic potentials in the entorhinal cortex evoked by stimulation of the piriform (olfactory) cortex, and to determine how synaptic responses are modulated by food-related cues.

**Results:**

The amplitudes of evoked synaptic responses were reduced during exposure to cues associated with delivery of chocolate, and during delivery of chocolate for consumption at unpredictable intervals. Reductions in synaptic responses were not well predicted by changes in behavioural mobility, and were not fully blocked by systemic injection of either the D_1_-like receptor antagonist SCH23390, or the muscarinic receptor antagonist scopolamine. However, the reduction in synaptic responses was blocked by injection of the D_2_-like receptor antagonist eticlopride.

**Conclusions:**

Exposure to cues associated with palatable food results in a suppression of synaptic responses in olfactory inputs to the entorhinal cortex that is mediated in part by activation of dopamine D_2_ receptors.

## Background

Dopaminergic projections to the entorhinal cortex are likely to play an important role in modulating the manner in which the entorhinal cortex contributes to sensory integration and mnemonic processing. The entorhinal cortex provides the hippocampus with much of its cortical sensory input and is thought to play roles in sensory integration, memory formation, and spatial navigation [1-4]. The lateral division of the entorhinal cortex receives heavy inputs from primary olfactory cortex suggesting that it plays an important role in olfactory processing and integrating olfactory information with other sensory inputs [1,5], and it also receives a large projection from midbrain dopamine neurons [6-11]. Dopaminergic effects on neuronal excitability (e.g.s, [12,13]), and on synaptic transmission within the entorhinal cortex, are therefore likely to have important effects on cognitive processes as animals engage in motivated behaviours associated with activation of dopamine neurons.

Application of dopamine to acute brain slices *in vitro* generally results in a *suppression* of glutamate-mediated synaptic responses in neurons in the entorhinal cortex that is mediated by activation of D_2_ receptors [5,14-16]. Strong dopaminergic input resulting in a suppression of glutamatergic synaptic transmission may interfere with cognitive processes [17], but a dopamine-mediated suppression of synaptic transmission might contribute to cognitive function by enhancing the signal to noise ratio for the most salient synaptic inputs [18], or by reducing interference of incoming synaptic inputs with ongoing mechanisms of working memory or long-term memory formation [19-22]. In addition, a relatively low concentration of 1 μM dopamine is known to result in a D_1_ receptor-mediated *facilitation* of synaptic responses in layer II of the entorhinal cortex *in vitro*[16], and systemic injection of a dopamine-reuptake blocker also facilitates entorhinal synaptic responses *in vivo*[5]. This raises the possibility that endogenous release of dopamine within the entorhinal cortex might facilitate the strength of synaptic inputs and thereby, enhance the impact or salience of reward-related stimuli.

The present study used palatable food reward as a stimulus to assess how the strength of synaptic inputs to the entorhinal cortex might be modulated following endogenous release of dopamine in response to anticipation and consumption of a natural reward. Exposure to food and food-conditioned cues activates dopamine neurons and results in increased dopamine in the nucleus accumbens and prefrontal cortex [23-30] and dopamine levels in the entorhinal cortex are also likely to be elevated [7,10]. Evoked synaptic field potentials were recorded in layer II of the lateral entorhinal cortex in response to stimulation of synaptic inputs from the piriform cortex using chronically implanted electrodes, and changes in the amplitudes of synaptic responses were assessed during intermittent delivery of small chocolate chips that served as a highly palatable food reward. Dopamine neurons are activated in response to reward-predictive cues [27,31-33], and we therefore also assessed how synaptic responses are modulated during exposure to cues previously associated with chocolate. The dependence of the resulting suppression effects on dopaminergic and cholinergic receptors was investigated using systemic injection of transmitter receptor blockers.

## Methods

### Surgery

Fifteen male Long-Evans rats (8 to 10 weeks old; Charles-River) were housed individually and had free access to food and water prior to behavioural testing, and experiments were conducted in accordance with the guidelines of the Canadian Council on Animal Care. Animals were anesthetised with isoflurane (1.5 to 2% in O_2_) and placed in a stereotaxic apparatus. A bipolar, twisted wire, Teflon-coated stainless-steel stimulating electrode (125 μm tips) was lowered into the right piriform cortex (P, 3.6 mm; L, 6.5 mm; V, 9.0 mm; tip separation of 1.0 mm) and a bipolar recording electrode was lowered into the lateral entorhinal cortex (P, 6.5 mm; L, 6.5 mm; V, 7.5 to 8.5 mm; tip separation of 0.8 mm). Vertical positions of electrodes were adjusted to maximize the amplitude of evoked field excitatory postsynaptic potentials (fEPSPs). A jeweler’s screw in the contralateral frontal bone served as a reference electrode, and a screw in the left parietal bone served as ground. Electrode leads were mounted in a plastic 9-pin connector and the assembly was fixed to the skull by embedding the jeweler’s screws, electrodes, and connector in dental cement. Buprenorphine (0.02 mg/kg, s.c.) was administered following surgery. There was a two-week recovery period with free access to food following surgery. Beginning 1 week prior to testing, access to food was restricted [34] to 45 g/day such that they maintained 90% of their free-feeding body weight.

### Conditioning and Field Potential Recordings

Animals were tested in a 40 × 40 × 60 cm Plexiglas chamber with a wire-grid floor surrounded by a Faraday cage. A light cue (37 lumens) was placed on top of the door. A 2.5 cm-diameter opening in the door allowed an aluminum tube (2 cm diameter) to be used to manually deliver chocolate chips (0.11 g) to a removable steel feeding cup (4 cm high, 6 cm diameter) affixed to the floor 4 cm from the door.

Each rat underwent five days of preliminary training to associate the feeding cup, aluminum tube, and cue-light with delivery of chocolate. On each day there were two 15-min counterbalanced sessions, one in which they received chocolate in the presence of the cue-light, feeding cup and delivery tube, and one in which these cues were absent and chocolate was not delivered. In order to deliver chips unpredictably [27] with an average of 1 chip per minute, intervals between chips were obtained from a lagged exponential distribution with a mean interval of 45 sec (maximum 120 sec) plus a 15 sec lag to allow time for consumption of each chocolate chip. Fourteen intervals were sampled at even steps across the probability distribution and rats obtained 15 chips within the 15 min period at intervals of 18 to 135 sec.

Field potential recordings were used to assess the effects of exposure to chocolate-related cues and consumption of chocolate on synaptic responses in the entorhinal cortex. A computer digital-analog channel and a stimulus isolation unit (A-M Systems, Model 2200) were used to deliver 0.1 ms biphasic constant-current square-wave pulses to the piriform cortex. A stimulus intensity that evoked synaptic responses that were 60 to 75% of maximal was determined for each animal (400 to 800 μA; mean, 576 μA). Evoked synaptic potentials were filtered (0.1 Hz to 5 kHz pass-band), amplified (A-M Systems, Model 1700), and digitized at 10 kHz (12-bit) for storage on computer hard disk using the software package Sciworks (Datawave Tech.).

Beginning the day following the completion of training, testing sessions were conducted every 2 days following systemic injection of either saline or a receptor blocker. On each day, synaptic responses were recorded every 15 sec during four consecutive time periods. In a subset of 9 animals tested, the cumulative time that the animal spent immobile in these periods (all four limbs in contact with the floor with no limb, neck or jaw movement) was recorded using a stopwatch. There was a 15 min baseline period in the absence of the cue-light and feeding cup. This was followed by the cued period in which the cue-light was on and the feeding cup was introduced for 10 min, but no chocolate was delivered. The 10-min chocolate-consumption period began with delivery of a chocolate chip to the feeding cup, and animals were allowed to consume a total of 10 chips delivered at unpredictable intervals taken from the lagged exponential distribution described above (mean interval of 45 sec plus 15 sec for consumption; range 17 to 135 sec). The cue light was turned off and feeding cup removed during the 15-min duration follow-up period. Tests on the first day were always conducted following a control injection of saline (0.9%; 1 ml/kg, i.p.) 15 min prior to recordings. The second and third tests were counterbalanced, and were conducted 15 min following administration of either the D_1_ antagonist SCH23990 (0.05 mg/kg, i.p.) or the D_2_ antagonist eticlopride (0.1 mg/kg, i.p.). A final test was conducted following injection of the muscarinic receptor antagonist scopolamine (3 mg/kg, i.p.). Peak amplitudes of averaged evoked field potentials were measured relative to the pre-stimulus baseline using SciWorks software. Planned comparisons (modified Bonferroni) [35] were used to assess changes in the amplitude of responses associated with cues or consumption of chocolate relative to the baseline period of each testing session, and to assess the reversal of changes in responses during the follow-up period (Statistica; Statsoft, Inc).

## Results

Animals readily consumed the food reward during preliminary training to associate chocolate with the cue-light and feeding cup. Between 2 and 8 of the 15 chips presented during the first testing session were consumed, and all animals consumed all chips presented in subsequent sessions.

**Figure 1:**
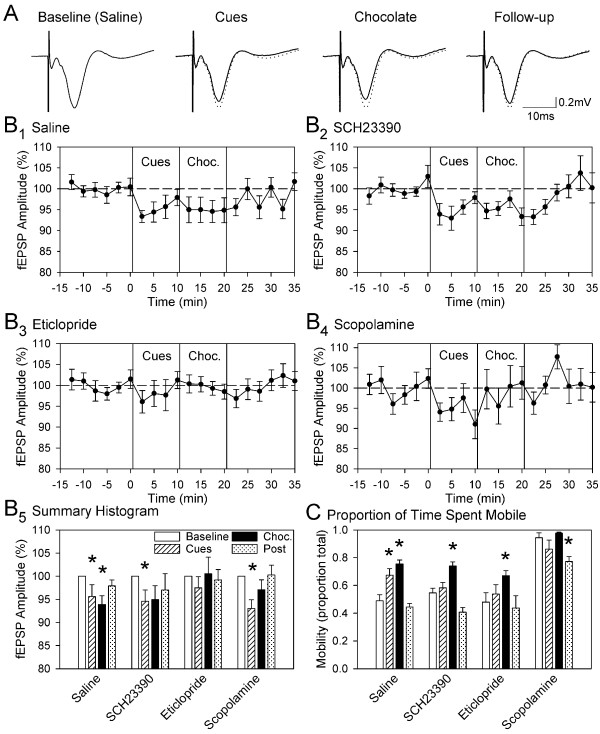
**The presentation of cues associated with chocolate, and the intermittent delivery of chocolate chips, results in a reduction in the amplitude of synaptic responses in the entorhinal cortex evoked by piriform cortex stimulation.** Synaptic responses were evoked every 15 sec during a baseline period, a cued period in which a cue-light and a feeding cup associated with chocolate were presented, a consumption period in which animals obtained 10 chocolate chips (1.1 g total) over a ten-min period, and a follow-up period without cues or chocolate. **A**: Averaged evoked synaptic field potentials from a representative animal are shown for each testing period following an injection of saline. The averaged baseline trace is superimposed as dotted lines for comparison. **B**: Mean amplitudes of field excitatory postsynaptic potentials (fEPSPs) averaged in 2.5 min-duration bins and are shown for tests conducted following injection of saline (B_1_, n = 14), the D_1_ receptor blocker SCH23990 (B_2_), the D_2_ receptor blocker eticlopride (B_3_), or the muscarinic receptor blocker scopolamine (B_4_). Data for each animal have been normalized to the mean amplitude of responses during the baseline period, and error bars indicate ± one standard error of the mean. Mean results are compared across testing conditions in B_5_ (*p < .05 vs. baseline). **C**: The mean proportion of time that animals spent moving during each phase of testing reflects increased mobility during anticipation and consumption of chocolate following saline injections, increased mobility during consumption of chocolate following injection of either D_1_ or D_2_ receptor antagonists, and increased mobility following injection of scopolamine.

To assess the effects of anticipation or consumption of chocolate on the strength of synaptic inputs to the entorhinal cortex, the amplitudes of synaptic potentials evoked by stimulation of the piriform cortex were contrasted between the baseline period, a "cued" period in which animals were exposed to a light cue and feeding cup associated with chocolate, a chocolate-consumption period, and a follow-up period in which cues associated with feeding were removed (Figure 1A, B_1_). In animals injected with saline, the amplitude of evoked potentials was reduced to 95.6 ± 2.6% of baseline values during the cued period (0.55 ± 0.08 vs. 0.58 ± 0.08 mV; n = 14, F_1,13_ = 4.70, p < .05) and to 93.9 ± 1.9% of baseline values during the consumption period (0.54 ± 0.08 mV; F_1,13_ = 10.77, p < .01). Responses during the follow-up period showed a partial reversal and did not differ significantly from baseline values (97.9% of baseline, p = 0.12). The presentation of cues that predict delivery of chocolate, as well intermittent consumption of chocolate, are therefore both associated with a moderate suppression of synaptic strength within the entorhinal cortex.

Tests on separate days were used to determine if systemic injection of D_1_- or D_2_-like receptor antagonists might block changes in evoked responses associated with exposure to chocolate. When animals were pre-injected with the D_1_-like receptor blocker SCH23990, the mean amplitude of synaptic responses was suppressed during both the cued period and the consumption period, but only the suppression during the cued period was statistically significant (Figure 1B_2_; cued, 94.6 ± 2.4%, F_1,13_ = 4.93, p < .05; consumption, 95.0 ± 3.0, F_1,13_ = 2.87, p = .11). However, administration of the D_2_ receptor blocker eticlopride prevented significant reductions in synaptic responses during both the cued and consumption periods (Figure 1B_3_; cued, 97.5 ± 2.4%, F_1,13_ = 1.08, p = .32; consumption, 100.6 ± 3.6%, F_1,13_ = 0.02, p = .87), suggesting that activation of D_2_ receptors is required for the suppression effect. Additional statistical comparison of the saline and eticlopride conditions showed that 2 by 2, drug by session, interactions were significant for the consumption period (F_1,13_ = 5.76, p < .05) but not for the cued period (F_1,13_ = .44, p = .51) indicating a more reliable effect of eticlopride during the consumption period, and suggesting in incomplete block of the suppression by eticlopride during exposure to cues. The analagous interaction comparisons were not statistically significant for SCH23390 for either the cued (F_1,13_ = .23, p = .64) or consumption (F_1,13_ = .18, p = .68) periods.

The amplitude of evoked synaptic responses in the entorhinal cortex can be attenuated by activation of muscarinic acetylcholine receptors *in vivo* during periods of movement (Hamam et al., 2007). Tests were therefore repeated following systemic injection of the muscarinic receptor blocker scopolamine. Reductions in synaptic responses associated with exposure to food-related cues were maintained in the presence of scopolamine (93.0 ± 1.9%, F_1,13_ = 13.59, p < 0.01) but there reduction observed during the consumption period was not statistically significant (97.1 ± 2.1%, F_1,13_ = 1.87, p = 0.19), suggesting that muscarinic receptors may contribute to the suppression observed during the consumption period (Figure 1B_4_).

Analysis of the proportion of time spent mobile during each of the testing phases revealed several effects (Figure 1C). First, there was a significant and reversible increases in mobility in the cued and consumption periods relative to the baseline period following saline injection (F_1,7_ = 28.07, p < 0.01, and F_1,7_ = 30.02, p < 0.001, n = 8, respectively) suggesting that exposure to cues led animals to anticipate delivery of chocolate. Second, there were significant increases in mobility during the consumption period following injections of either SCH23390 or eticlopride (F_1,7_ = 10.46, p < 0.05 and F_1,7_ = 10.66, p < .05 respectively), and smaller changes in movement during the cued period (F_1,7_ = 0.63, p = 0.45 and F_1,7_ = 7.38, p < 0.05, respectively), suggesting that dopamine blockers may have reduced mobility associated with anticipation of reward or that animals had learned by the second and third testing days that chocolate was not immediately forthcoming. Third, there was increased mobility during baseline following injection of scopolamine in comparison to the saline condition (F_1,7_ = 291.3, p < .001) consistent with the behavioural activating effects of that drug [36].

## Discussion

In the experiments described here we have used a palatable food reward to assess the potential modulatory effect of a rewarding stimulus, and reward-related cues, on synaptic transmission in the entorhinal cortex. Results show that both the exposure to cues associated with chocolate, and the intermittent delivery of chocolate chips for consumption, results in a reversible suppression of the strength of synaptic responses in the lateral entorhinal cortex evoked by piriform cortex stimulation. The size of the suppression effect was relatively small - about 5% of baseline values - but the reliability of the effect allowed pharmacological tests of the contributions of dopamine receptor subtypes and muscarinic receptors. The suppression of evoked synaptic responses during exposure to food-related cues and during the consumption period was dependent upon activation of D_2_ dopamine receptors, and this suggests that increases in dopamine release associated with natural rewards can result in a suppression of glutamatergic synaptic inputs [16] to the entorhinal cortex. We have observed stronger synaptic suppression effects of 11% in the same preparation in association with rewarding electrical stimulation of the hypothalamus [37], and 43% following direct application of 100 μM dopamine to the entorhinal cortex in vitro [5,16], but the smaller synaptic suppression induced here by endogenous release of dopamine associated with food-related cues is likely to reflect important effects of dopamine on reward-related synaptic processing within the entorhinal cortex.

The D_2_ receptor antagonist eticlopride was effective in blocking the suppression of synaptic responses induced by food-related cues and by chocolate, but other neuromodulatory transmitters that modulate synaptic responses in the entorhinal cortex may be active during anticipation and consumption of food reward [30,38-41]. Acetylcholine release during periods of mobility suppresses glutamate-mediated synaptic transmission in the entorhinal cortex via activation of muscarinic receptors [42-45]. Although the muscarinic receptor antagonist scopolamine did not prevent the suppression of evoked responses during exposure to food-related cues, the reduction in responses during delivery of chocolate was not significant (Figure 1B_4_), suggesting that muscarinic receptors may play a role. Increased behavioural activity can increase hippocampal EPSP slope in part due to increased brain temperature [46,47] but similar brain-temperature dependent effects are unlikely to contribute to the *suppression* of responses induced here.

The finding that the suppression of synaptic responses is associated with the anticipation of food reward, as well as with the intermittent delivery of chocolate chips, is consistent with the ability of reward-predictive cues to activate dopamine neurons and to modulate cortical excitability [32,48]. The anticipation of food reward induces strong elevations in dopamine in the nucleus accumbens and prefrontal cortex, followed by declines during consumption [25-28,34,49], and exposure to food reward results in changes in cortical activation in humans in areas including the striatum, insula, orbitofrontal, pregenual and parahippocampal cortices [50-53]. Results obtained here indicate that exposure to food-related cues can reduce overall strength of olfactory inputs to the entorhinal cortex. The D_2_ receptor-dependent reduction in glutamate-mediated synaptic responses induced by chocolate is consistent with previous findings *in vitro* that have shown that, although low concentrations of dopamine can facilitate synaptic responses via D_1_ receptors, higher concentrations of dopamine mediate a suppression of synaptic transmission primarily via activation of D_2_ receptors [14-16]. The block of the synaptic suppression by eticlopride in the present study is therefore consistent with the known effects of D_2_ receptors in the entorhinal cortex but, because eticlopride was administered systemically, the relevant D_2_ receptors could be elsewhere; dopamine D_2_ receptor antagonism might also block the suppression effect by reducing reward-induced activation of brain regions by dopamine [52]. In addition, strong D_1_ receptor activation can lead to a suppression of entorhinal synaptic response by affecting cellular input resistance [16], and it is possible that a higher dose of SCH23390 might reveal a possible contribution of D_1_ receptors to the synaptic suppression.

It is possible that the absence of significant reductions in synaptic responses during consumption of chocolate during drug tests might be due to a learning or experience-induced change, rather than due to drug administration, because the saline condition was always tested first prior to counterbalanced tests of the effects of D_1_ and D_2_ receptor blockers. Suppression effects observed during exposure to cues, in which there were significant reductions in the presence of SCH23390, and in final tests with scopolamine, suggest that carry-over effects may be minimal, but it is possible that the suppression of synaptic responses induced during intermittent consumption of food reward may be reduced over multiple testing sessions.

The synaptic suppression may weaken the functional impact of sensory inputs to the entorhinal cortex, but it might also serve to make active synaptic inputs relatively more salient in comparison to reduced spontaneous synaptic activity [18], or may reduce possible interference between extrinsic and intrinsic synaptic inputs in the service of protecting working memory representations or contributing to the storage of longer lasting representations [13,19-22,54]. In addition, activation of D_1_ receptors with *low* concentrations of applied dopamine *strengthens* synaptic responses *in vitro*[5,16], suggesting that dopamine may at times facilitate the strength of olfactory inputs to the entorhinal cortex. The suppression observed here was seen during constant exposure to food-predictive cues and during unpredictable delivery of chocolate chips, so that the evoked synaptic responses (that were recorded every 15 sec) were not closely related to temporally discrete food-related stimuli or operant responses; the suppression is therefore likely due to increased tonic levels of dopamine as opposed to shorter lasting phasic increases that can be evoked by reward-related cues [32,33]. Tests for the modulation of evoked responses at short intervals surrounding presentation of discrete food-related cues or performance of an operant response to obtain food reward might be used to investigate a possible phasic facilitation or suppression effect.

Additional factors that modulate dopamine release are likely to affect the suppression of synaptic strength observed here. Avena et al. [55] found that delivery of sucrose increased dopamine release in the nucleus accumbens of food-restricted rats, but not in non-food-restricted animals. We found here that, although allowing animals free access to lab chow did not prevent the suppression of synaptic responses evoked by exposure to cues (94.5 ± 1.8%, p <0.05), it did prevent reductions in responses during consumption of chocolate (data not shown); the maintained reduction in synaptic responses during exposure to cues may reflect the power of cues to activate dopamine neurons, particularly during anticipation of initial consumption of the palatable food reward and, similar to the findings of Avena et al. [55], the lack of a reduction in synaptic responses during consumption in free-feeding animals is likely to be associated with a reduced value of the reward through a reduction in motivational state. Similar manipulations of variables including motivational state, reward value (e.g., regular versus palatable food), predictor cues, and the costs and/or predictability of obtaining rewards through different reinforcement schedules might be used to investigate the variables that determine changes in cortical excitability associated with food reward [24,26,31,33,34].

## Competing interests

The authors declare that they have no competing interests.

## Authors’ contributions

CAC conceived the study and JAH collected the data. Both authors contributed to the design of the study, data analysis, and preparation of the manuscript. Both authors read and approved the final manuscript.
